# Candidalysin biology and activation of host cells

**DOI:** 10.1128/mbio.00603-24

**Published:** 2025-04-28

**Authors:** Léa Lortal, Claire M. Lyon, Jakob L. Sprague, Johannes Sonnberger, Olivia K. A. Paulin, Don N. Wickramasinghe, Jonathan P. Richardson, Bernhard Hube, Julian R. Naglik

**Affiliations:** 1 Centre for Host-Microbiome Interactions, Faculty of Dentistry, Oral and Craniofacial Sciences, King’s College London, London, United Kingdom; 2 Department of Microbial Pathogenicity Mechanisms, Leibniz Institute for Natural Product Research and Infection Biology–Hans Knöll Institute (HKI), Jena, Germany; 3 Institute of Microbiology, Friedrich Schiller University, Jena, Germany; 4 Cluster of Excellence Balance of the Microverse, Friedrich Schiller University Jena 9378, Jena, Germany; Instituto Carlos Chagas, Curitiba, Brazil

**Keywords:** *Candida albicans*, candidalysin, fungal infection, immune mechanisms, toxins

## Abstract

*Candida albicans* is an opportunistic fungal pathogen that can cause life-threatening systemic infections and distressing mucosal infections. A major breakthrough in understanding *C. albicans* pathogenicity was the discovery of candidalysin, the first cytolytic peptide toxin identified in a human pathogenic fungus. Secreted by *C. albicans* hyphae and encoded by the *ECE1* gene, this 31-amino acid peptide integrates into and permeabilizes host cell membranes, causing damage across diverse cell types. Beyond its cytolytic activity, candidalysin can trigger potent innate immune responses in epithelial cells, macrophages, and neutrophils. Additionally, candidalysin plays a key role in nutrient acquisition during infection. This review explores the biology of candidalysin, its role in host cell activation, and extends the discussion to non-candidalysin Ece1p peptides, shedding light on their emerging significance.

## INTRODUCTION

Fungal infections are a major threat to global health, affecting over 1 billion people worldwide and killing 3.8 million people every year (1–3). Alarmingly, fungal infections kill almost three times more individuals than tuberculosis and six times more than malaria (World Health Organization, 2022). Despite these high numbers, fungal diseases remain largely neglected and understudied.

Fungal infections can manifest as superficial (e.g., nails and skin), mucosal (e.g., oral and vaginal), or invasive (e.g., bloodstream and organs). Invasive fungal infections affect over 6.5 million people per year and have an alarming average mortality rate of 50% (1, 2). Among the most common pathogens responsible for mucosal and systemic fungal infections is *Candida albicans. C. albicans* is a normal component of the human microbiota, commonly colonizing the mouth, gastrointestinal tract, and vagina of healthy individuals (4–6). However, under certain conditions, this opportunistic pathogen can proliferate and cause distressing mucosal infections, such as vulvovaginal candidiasis (VVC) and oral candidiasis. Notably, approximately 75% of women experience VVC at least once in their lifetime (7, 8), and recurrent VVC (RVVC; defined as four or more VVC episodes per year) affects 138 million women annually (8, 9). Oral candidiasis further burdens global health, affecting 15 million people each year (1). In addition to mucosal infections, *C. albicans* is a leading cause of invasive candidiasis, one of the most prevalent and life-threatening invasive fungal infections globally (1, 10).

Current medications for mucosal infections are relatively effective but are not preventive. Thus, there is currently no adequate treatment against RVVC. Risk factors for mucosal infections include pregnancy, immunosuppression, genetic predispositions, and use of antibiotics, glucocorticoids, and oral contraceptives (4, 6, 7).

*C. albicans* virulence attributes include morphological transitions, factors facilitating adhesion and invasion, the production of hydrolytic enzymes, biofilm formation, escape from phagocytosis, and evasion from the host immune system (11, 12). A fundamental advance in understanding the pathogenicity of *C. albicans* was the discovery of candidalysin, a cytolytic peptide toxin secreted by *C. albicans* hyphae (13). Candidalysin is the first cytolytic peptide toxin to be identified in any human pathogenic fungus. This 31-amino acid peptide intercalates and permeabilizes host cell membranes, causing damage across various cell types. Through its activity, candidalysin also triggers robust innate immune responses in the host (13, 14). In recent years, there has been a plethora of studies on candidalysin and how the toxin drives infection and immune responses in several different model systems. As such, this review will focus predominantly on candidalysin biology and its role in activating host cells during infection. The reader is guided to several other reviews that focus on the role of candidalysin in driving downstream immune responses and other disease states (14–19).

## CANDIDALYSIN PRODUCTION AND SECRETION

The success of *C. albicans* both as a commensal and pathogen resides in its remarkable capacity to adapt to different host niches and environmental cues. Such adaptability is most evident in the context of morphological plasticity at mucosal surfaces. Indeed, *C. albicans* is a polymorphic fungus that can grow as ovoid-shaped yeasts, pseudohyphae, and hyphae (20). Morphological transitions are a key virulence factor, as hyphae can breach epithelial barriers and penetrate underlying tissues and are, therefore, synonymous with mucosal infections (21–25). However, the yeast form has also been associated with dissemination, highlighting the importance of morphological plasticity (11).

The yeast-to-hypha transition can be induced by several factors, including endogenous cues, such as cell cycle progression, and environmental stimuli, such as exposure to serum, temperature (37°C), 5% carbon dioxide, neutral pH, and the presence of N-acetylglucosamine (26–29). The hyphal morphology is regulated by three main signal transduction pathways: the cAMP-PKA (cyclic adenosine monophosphate-protein kinase A) pathway, which targets the transcription factor enhanced filamentous growth protein 1 (Efg1), the Cek1 MAPK (extracellular signal-regulated kinase mitogen-activated protein kinase) pathway, and the pH response pathway (27, 30). Signaling through these pathways converges on transcriptional activators, including Cph1p, Flo8p, Ume6p, and Tec1p, which control the expression of hypha-associated genes (31, 32). Cell density and interactions with other microorganisms via quorum sensing molecules, such as farnesol, can also induce transitions from one morphology to the other, which activates transcriptional repressors, such as Nrg1p, Rfg1p, and the co-repressor Tup1p which downregulate the expression of hypha-associated genes (11, 27–29).

A core filamentation response network comprising only eight genes was originally identified (33), which is upregulated following a change in pH (from pH 4 to 8), in the presence of 10% human B serum and following a change in carbon source (from glucose to N-acetylglucosamine) and was expanded recently (34). This network includes genes whose proteins have known (or likely) cell wall-associated functions (*ALS3*, *HGT2*, *HWP1*, *IHD1*, *RBT1*), *DCK1* (a putative guanine nucleotide exchange factor), orf19.2457 (an ORF of unknown function), and *ECE1*.

Candidalysin is encoded by the Extent of Cell Elongation 1 (*ECE1*) gene, which is strongly upregulated during hyphal growth (13, 33, 35). Although strongly expressed in hyphae, Ece1p is not required for hyphal growth (13, 35) but instead plays a crucial role in virulence (13). Truncation analysis of the 3,197 bp *ECE1* intergenic region demonstrated that the *ECE1* promoter has a minimal size of 1,500 bp and a TATA element located 106–109 nucleotides upstream of the start codon (36). The presence of numerous transcription factor binding sites in the *ECE1* promoter suggests that the regulation of *ECE1* expression is likely a complex event. However, so far, only Ahr1p and Tup1p have been shown to play a direct role in *ECE1* regulation (37).

**Fig 1:**
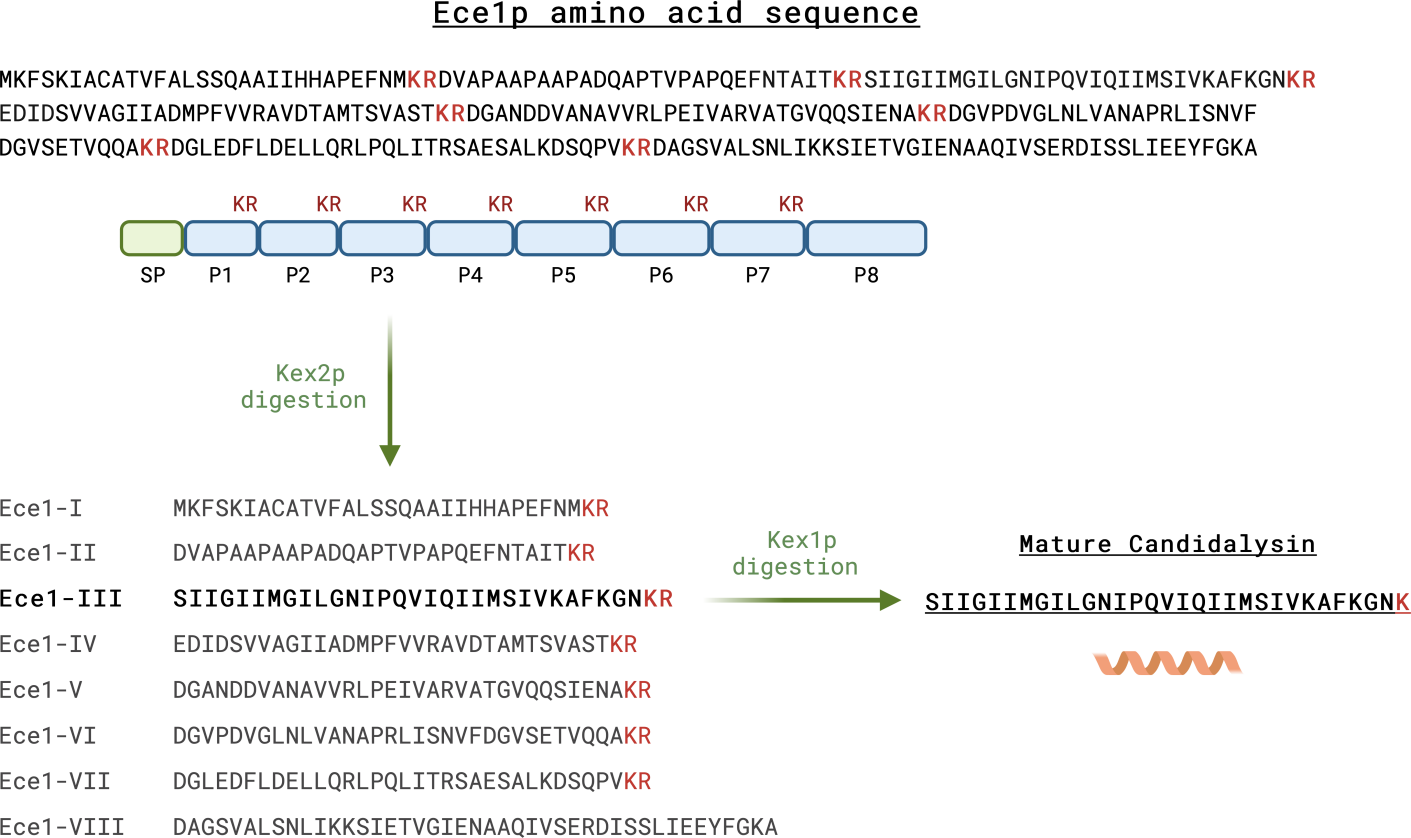
Candidalysin generation by *C. albicans*. The *ECE1* gene is strongly expressed during *C. albicans* hyphal growth and encodes Ece1p, a polypeptide of 271 amino acids. The polypeptide is sequentially processed, first by Kex2p at lysine-arginine (KR) motifs (in red) to produce eight peptides, and then by Kex1p, which cleaves the terminal arginine residues. Candidalysin is the third peptide (position 62–92) and is 31 amino acids in length. SP = signal peptide. Created with BioRender.com.

Following expression, *ECE1* mRNA transcripts are translated, and Ece1p transits through the fungal endomembrane system. The Ece1p pre-pro-protein contains 271 amino acids. A notable feature of Ece1p is the presence of seven dibasic lysine-arginine (KR) motifs dispersed throughout the protein (35). Ece1p is processed sequentially by two kexin proteinases: first by the endoproteinase Kex2p, which cleaves Ece1p after each KR motif to produce seven short peptides and an immature candidalysin toxin containing a C-terminal arginine (SIIGIIMGILGNIPQVIQIIMSIVKAFKGNK**R**), and then by the carboxypeptidase Kex1p, which removes the C-terminal arginine to produce the mature toxin terminating in lysine. Candidalysin is the third peptide (amino acid positions 62–92; SIIGIIMGILGNIPQVIQIIMSIVKAFKGNK) (Fig. 1) (13, 38, 39). Efficient processing of *C. albicans* Ece1p after Arg61 and Arg93 is critical for pathogenicity, as alanine substitution at these positions attenuates virulence *in vivo* (39). Indeed, natural variations in the amino acid sequence of the candidalysin P2–P3-processing site within Ece1p have a profound influence on the efficiency of kexin-mediated processing and, hence, candidalysin production, which in turn has a direct impact on virulence *in vivo* (40).

The secreted candidalysin peptide is amphipathic and α-helical and resembles catanionic antimicrobial peptides and peptide toxins, such as melittin and alamethicin (13). A recent study has shown that candidalysin pre-assembles in solution into polymer loops of several 8-mers, which then intercalate into the membrane, forming large, cyclo-octameric permeabilizing pores (41).

## CANDIDALYSIN AND EPITHELIAL CELLS

**Fig 2:**
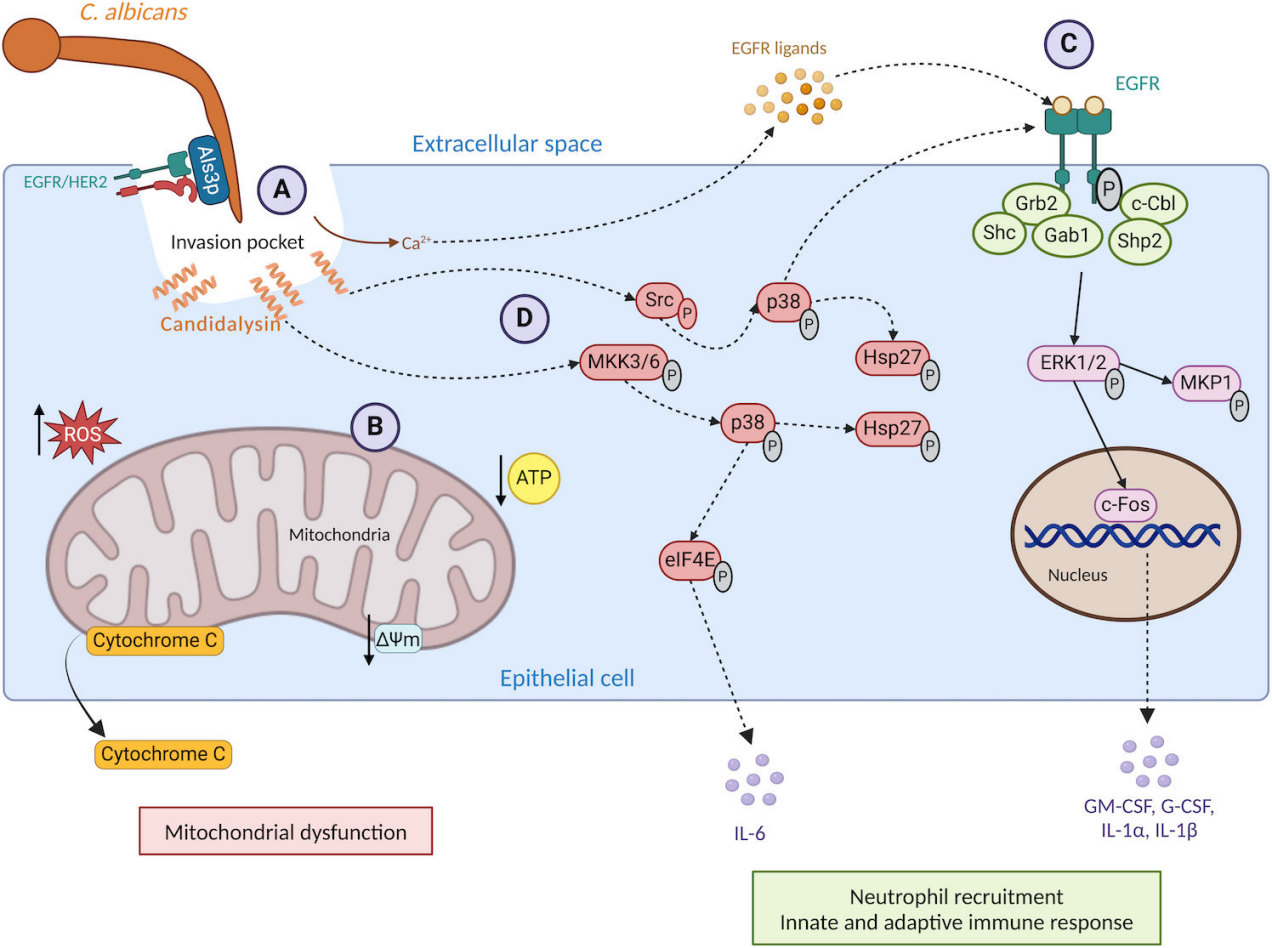
Candidalysin activates epithelial cells. *C. albicans* hyphae are recognized by epithelial cells through the binding of host surface proteins, such as EGFR/HER2, to invasins and adhesins like Als3p, creating the invasion pocket (A). Candidalysin is secreted by hyphae and accumulates in the invasion pocket. It forms pores in the plasma membrane of epithelial cells, triggering calcium influx and LDH release. Candidalysin induces mitochondrial dysfunction by reducing intracellular ATP, reducing mitochondrial membrane potential (ΔΨm), increasing intracellular reactive oxygen species (ROS), and promoting the release of cytochrome c into the extracellular environment (B). Calcium influx stimulates the activation of matrix metalloproteinases, which release surface-tethered epidermal growth factor receptor (EGFR) ligands. These ligands bind to EGFR, triggering receptor dimerization and activation (C). EGFR activation stimulates ERK1/2, which activates the MAPK phosphatase MKP1. The transcription factor c-Fos is then activated, leading to the expression of cytokines (G-CSF, GM-CSF, IL-1α, and IL-1β), which are released from the cell and stimulate neutrophil recruitment and type 17 immunity at the site of infection. In parallel, candidalysin activates the p38-MAPK pathway, which can also activate EGFR and Hsp27 and stimulate IL-6 secretion (D). Created with BioRender.com.

The mucosal epithelium is a key site of *C. albicans* colonization and infection and is the first line of defence, providing a physical barrier against tissue invasion. Adhesion to epithelial cells is an essential pre-requisite for commensal colonization and pathogenic invasion (23, 42). *C. albicans* hyphae express numerous adhesins, including agglutinin-like sequence 3 (Als3p) and hyphal wall protein 1 (Hwp1p). Als3p binds to epithelial receptors, such as E-cadherin, whereas Hwp1p covalently bonds to the cell after being processed by host transglutaminases (43–46). Once adhered, hyphae can invade epithelial cells by two distinct and complementary mechanisms: active penetration (AP) and receptor-induced endocytosis (RIE) (45, 47, 48). AP is the primary mode of invasion, whereby extending hyphae physically exert force onto the host cell while secreting hydrolytic enzymes (48). These enzymes include phospholipases, lipases, and secreted aspartyl proteases, which can degrade host membrane phospholipids, triglycerides, and peptides (49). Conversely, RIE is a host-driven process, whereby the epidermal growth factor receptor (EGFR) and Her2 on the host cell surface interact with fungal-surface invasins Als3p and Ssa1p, triggering phosphorylation events, which stimulate endocytosis (50, 51). AP and RIE result in the formation of an invasion pocket (Fig. 2A), in which the invading hyphal tip is encased within the invaginated host cell membrane (52). Candidalysin is secreted into this invasion pocket, where it accumulates and then intercalates into the host cell membrane, forming pores (41, 52). This destabilizes the host plasma membrane and leads to the release of cellular contents, including lactate dehydrogenase (LDH), antimicrobial peptides, and alarmins (13, 14, 53), ultimately inducing cell death. Interestingly, candidalysin does not cause cell death by apoptosis, as programmed cell death markers, such as activation of caspases 3 or 8 or cleavage of poly(ADP-ribose) polymerase, are not reported to occur (54). However, candidalysin induces rapid mitochondrial dysfunction by reducing intracellular ATP, reducing mitochondrial membrane potential (ΔΨm), increasing intracellular reactive oxygen species (ROS), and promoting the release of cytochrome c into the extracellular environment (Fig. 2B). Collectively, these data demonstrate that cell death occurs through a necrotic mechanism (54). Additionally, ΔΨm depolarization does not take place in the absence of extracellular calcium, indicating calcium influx is important for mitochondrial dysfunction (54). It is well reported that candidalysin causes the rapid influx of extracellular calcium ions into epithelial cells *in vitro* (13, 54–56). Candidalysin-induced calcium influx has been quantified in epithelial cells by whole-cell patch clamping, Fura-2 binding (13), and by using genetically encoded fluorescent calcium sensors (GCaMP6s) (56). The use of GCaMP6s demonstrated that transient spikes in the level of intracellular calcium occurred up to once every 5 min, returning to baseline in between spikes. Calcium spikes coincided with plasma membrane blebbing (visible within 10 min), in which candidalysin-containing membrane was “pinched off” from the treated cell and released into the extracellular environment. Notably, blebbing required components of the cellular repair complex, including apoptosis-linked gene 2 (ALG-2), ALG-2-interacting protein X (ALIX), and “endosomal sorting complex required for transport-III” (ESCRT-III), suggesting calcium influx may contribute to the host’s attempt to remove candidalysin and repair membrane damage (56).

In addition, in oral epithelial cells, calcium influx also indirectly activates matrix metalloproteinases, which cleave surface-tethered ligands, such as epigen, epiregulin, and amphiregulin. These ligands in turn bind and activate EGFR (Fig. 2C) (55). EGFR is a key receptor in mammalian cells, regulating numerous signalling pathways involved in cell growth, survival, division, differentiation, angiogenesis, and migration. EGFR activation by candidalysin triggers the extracellular signal-regulated kinase 1/2 (ERK1/2) MAPK pathway, leading to the expression and phosphorylation of the MAPK phosphatase MKP1 (14, 55, 57, 58). The ERK1/2 pathway also induces the expression of the transcription factor c-Fos, driving the production of immune-modulatory cytokines, such as granulocyte colony-stimulating factor (G-CSF), granulocyte-macrophage colony-stimulating factor (GM-CSF), and interleukins 1α and β (IL-1α and IL-1β). Simultaneously, candidalysin also activates the p38 signalling pathway through two independent routes: MKK3/6 and proto-oncogene tyrosine-protein kinase Src (Fig. 2D). MKK3/6 phosphorylates p38, ultimately leading to the production of interleukin 6 (IL-6), while Src also phosphorylates p38 but instead triggers EGFR activation in a ligand-independent manner (59). Both routes activate heat shock protein 27 (Hsp27) (59), but the function of this protein during infection is unknown. The release of cytokines induces neutrophil recruitment and type 17 immunity; both crucial for host protection against oral candidiasis (13, 14, 55, 59, 60). Furthermore, IL-1α release triggers an early innate immune response in nearby epithelial cells via the IL-1 receptor (IL-1R) and NF-κβ signalling, leading to the production of additional pro-inflammatory cytokines and chemokines (61).

Candidalysin also activates five EGFR adaptors in oral epithelial cells: Grb2-associated-binding protein 1 (Gab1), growth factor receptor-bound protein 2 (Grb2), Src homology two and collagen protein (Shc), SH2-containing protein tyrosine phosphatase-2 (Shp2), and casitas B-lineage lymphoma (c-Cbl). These adaptors regulate ERK1/2-MAPK signaling and are shown to influence cytokine secretion when inhibited using small interfering RNA (62). Furthermore, during infection, EGFR and the ephrin type-A receptor 2 (EphA2) form a complex and activate each other mutually. Candidalysin activates both receptors, stimulating pro-inflammatory cytokine and chemokine secretion, while *C. albicans* binding to the EphA2-EGFR complex prevents receptor degradation (63).

## CANDIDALYSIN AND NEUTROPHILS

Neutrophils are an important part of the innate immune system and play a key role in host defence against fungal infections (64). Candidalysin has been identified as an inducer of neutrophil infiltration, with studies demonstrating significantly reduced neutrophil recruitment in murine models of oropharyngeal candidiasis (OPC) and zebrafish models of mucosal infection following infection with *ece1*Δ/Δ (13). In vaginal epithelial cells, candidalysin also causes damage and pro-inflammatory cytokine release, such as G-CSF, GM-CSF, and IL-1β, all of which are strongly linked to neutrophil recruitment (65). Neutralization of candidalysin by specific nanobodies can block epithelial damage, pro-inflammatory cytokine release, and neutrophil recruitment (66). Likewise, in a murine model of VVC, significantly fewer neutrophils were recruited in response to *ece1*Δ/Δ or the candidalysin null strain *ece1*Δ/Δ *+ ECE1*_Δ184–279_ compared to wild-type *C. albicans*-infected mice (65). However, while neutrophil recruitment triggers protective immune responses in OPC, it drives pathology in VVC by causing acute inflammation and exacerbating symptoms (67). Indeed, polymorphonuclear neutrophils may have limited protective function during VVC and struggle to reduce fungal burdens at the vaginal mucosa (68, 69).

Similarly, candidalysin is required for systemic murine neutrophil recruitment, as infection with *ece1*Δ/Δ leads to significantly reduced secretion of neutrophil-recruiting chemokines (e.g., CXCL1 and CXCL8), as well as reduced renal neutrophil infiltration (70). Nonetheless, one study found that the survival rate of mice infected with candidalysin-deficient strains was 100% at day 7 compared to 25% for mice infected with candidalysin-producing strains. Interestingly, mice infected with candidalysin-deficient strains had a significant increase in fungal burden in the spleen, kidney, and brain compared to wild-type *C. albicans* (70). This suggests that during systemic infection, fungal burden can be decoupled from the severity of infection and that other virulence factors, such as candidalysin secretion, are more important for driving pathogenicity.

In the brain, candidalysin stimulates protective neutrophil recruitment, whereby the toxin engages CARD9-positive microglia through CD11b to induce the secretion of IL-1β and CXCL1. IL-1β and CXCL1 recruit CXCR2-expressing neutrophils, leading to fungal clearance (71, 72).

Candidalysin has also been linked to the formation of neutrophil extracellular traps (NETs) (73). NETs are an extracellular mechanism used alongside phagocytic neutrophil killing, which enhance fungal eradication in a NADPH oxidase-dependent manner. However, if released in excess, the pro-inflammatory function of NETs can negatively impact the host (74). In addition to NETs, microbial toxins can induce leucotoxic hypercitrullination of histones found in neutrophils, which can cause NET-like structures (NLS) to form. NLS act in a NADPH-independent manner and are more compact and less fibrous than NETs, but both have similar functions (75).

In a recent study, candidalysin-deficient strains were shown to induce fewer NETs than candidalysin-expressing strains. Synthetic candidalysin was found to act as a stimulus for leucotoxic hypercitrullination and, thus, NLS release; however, NET formation was unaffected. This candidalysin-induced NLS activation relied partly on NADPH oxidase-mediated ROS production, as well as peptidyl arginine deiminase 4 (PAD4)-mediated histone citrullination (73). Interestingly, NETs are also induced by various *C. albicans* isolates, including strain 101, which expresses very low levels of the *ECE1* gene (76). These findings suggest a potential decoupling of candidalysin from NETosis—a form of neutrophil cell death that results in the NET and NLS release (76). In another study, which examined catheter-associated biofilms under a total parenteral nutrition environment, *C. albicans* clinical isolates with low candidalysin secretion levels exhibited resistance to neutrophil-mediated biofilm clearance, with undetectable NETosis. Treatment with synthetic candidalysin induced NETosis and aided the removal of biofilms, suggesting the toxin can be downregulated to promote *C. albicans* persistence in intravascular catheters (77).

## CANDIDALYSIN AND MACROPHAGES

In addition to neutrophils, macrophages and dendritic cells are important first-line responders to *C. albicans* infection. The primary function of macrophages is to phagocytose fungal cells, containing them in the phagosome, which then matures and leads to the intracellular killing of the fungus. However, *in vitro* studies have shown that a portion of intracellular *C. albicans* cells can form hyphae in the phagosome within a few hours, leading to the production and secretion of candidalysin (78, 79). Furthermore, macrophages may also encounter non-phagocytosed filaments of *C. albicans*, resulting in extracellular exposure to candidalysin. In both cases, the toxin induces macrophage cell death (79, 80). Notably, intracellular production of candidalysin contributes substantially to *C. albicans* escape from macrophages (79).

Macrophages can undergo different forms of programmed cell death as a response to the intracellular proliferation of phagocytosed pathogens. These pathways are also activated by intracellular *C. albicans* cells (81), with pyroptosis being the most prominent pathway induced (82–84). Pyroptosis of macrophages is characterized by host cell activation via intracellular multi-protein pattern recognition receptors. An example is the activation of the NLRP3 inflammasome, which comprises NLRP3, ASC, and pro-caspase 1. Upon NLRP3 inflammasome activation, pro-caspase 1 is cleaved and activated, leading to the processing and activation of the pore-forming protein GSDMD and the cytokine IL-1β (85). IL-1β is then predominantly released through GSDMD pores (86). The expression, assembly, and function of the NLRP3 inflammasome require priming and an activation signal, which are both provided by *C. albicans* (80). Indeed, intra- and extra-cellular candidalysin can act as an activation signal for the NLRP3 inflammasome following priming by factors, such as LPS or β-glucan (80, 87). In primary dendritic cells lacking components of the NLRP3 inflammasome, IL-1β release was completely abolished after infection with either *C. albicans* or treatment with synthetic candidalysin (80). In contrast, LDH release from phagocytes remained unchanged, suggesting that candidalysin-mediated host cell damage can occur independently of host inflammasome signaling (80).

Since candidalysin plays a key role in complex cellular processes within the host, understanding its subcellular localization and function with spatial and temporal precision is of particular interest. Although *ECE1* is expressed in germinating *C. albicans* cells within the phagosome, candidalysin is dispensable for phagosomal escape, even under conditions of forced overexpression in a yeast-locked mutant (88). Given that membrane composition influences candidalysin’s activity (89), it is conceivable that the phagosome membrane is resistant to candidalysin-mediated damage, leading to its accumulation within the phagosome. Upon filament-induced rupture of the phagosome, candidalysin may leak into the cytosol, gaining access to the macrophage plasma membrane (88). As a host defence strategy, calcium-dependent lysosomal fusion is required to contain elongating *C. albicans* filaments in an intact phagosome (78). Over time, nearly all cellular lysosomes are integrated into this process. Inhibition of this mechanism results in premature and increased activation of the NLRP3 inflammasome, potentially driven by candidalysin’s release into the cytoplasm and extracellular space (78).

While candidalysin-supported escape from macrophages benefits *C. albicans*, the resulting production of IL-1β can have niche-specific consequences that can be both advantageous or detrimental to the host and the fungus (16).

## CANDIDALYSIN AND PLATELETS

In the lung and oral cavity, candidalysin also has an effect on platelets. In the lung, candidalysin was identified as a key driver of *C. albicans*-mediated allergic airway disease (90). Mice infected with an *ece1*Δ/Δ mutant display significantly less airway hyperresponsiveness, reduced recruitment of immune cells, such as macrophages and neutrophils, and reduced cytokine secretion compared to mice infected with wild-type *C. albicans*. The toxin activates platelets by binding to the GP1bα receptor and induces the release of Dickkopf 1 (Dkk-1), leading to Th2 and Th17 cell responses. In the oral cavity, candidalysin activates tongue megakaryocytes to release platelets with antifungal capacity. Infection with a candidalysin-deficient strain resulted in decreased expansion of megakaryocytes during oral infection. Direct interaction between platelets and neutrophils was required since neutrophil influx was prevented when mice were treated with an anti-PSGL-1 antibody (which blocks the primary ligand for the platelet surface protein P-selectin), while transfusion of platelets rescued the neutrophil defect (91).

## CANDIDALYSIN AND ITS BINDING PARTNERS

Candidalysin appears to have several modes of action on a given host cell. Biophysical analysis revealed that candidalysin can directly intercalate into synthetic dioleoylphosphatidylcholine plasma membranes *in vitro* and forms pores into host plasma membranes to lyse cells (13, 41). However, notably, candidalysin does not integrate into *C. albicans* membranes, suggesting specificity for human membranes (89). Candidalysin can also directly bind host proteins, such as GP1bα on platelets, which can drive *C. albicans*-mediated allergic airway disease through the release of Dkk-1 (90). Candidalysin can also bind the integrin CD11b on microglia, which leads to protective neutrophil responses during *C. albicans* cerebritis (72).

Importantly, given that candidalysin is a toxin and the only known damage-inducing factor *C. albicans* produces, it is also logical that the host targets candidalysin to prevent its activity. As such, albumin and heparin were recently identified as two host proteins that can neutralize candidalysin activity through hydrophobic interactions (92, 93). Furthermore, a recent study identified sulfated glycosaminoglycans (GAGs) on epithelial cells as critical targets of candidalysin (94). Disruption of specific genes involved in GAG biosynthesis conferred resistance to candidalysin-induced damage, and the use of GAG analogues like dextran sulfate was shown to protect cells and reduce tissue damage and inflammation in murine models of vulvovaginal candidiasis.

Additionally, a recent study employing global fungal-host interactome mapping identified host protein cyclin H as a direct binding partner of candidalysin (95). This interaction activates the cyclin-dependent kinase-activating kinase, inhibiting the DNA damage repair pathway, thereby contributing to host cell damage during infection.

## CANDIDALYSIN AS A HEMOLYTIC FACTOR

Numerous studies dating back to the 1950s have shown that *C. albicans* displays hemolytic activity (96–99). Red blood cells represent a rich iron reservoir for *C. albicans*, which faces substantial iron limitation during systemic infection (100, 101). Notably, *C. albicans* has developed a specialized pathway for acquiring heme and iron from hemoglobin, a process that is limited to its hyphal morphology (98, 102). Initially, the hemolytic factor was believed to be a sugar moiety of a fungal mannoprotein; however, the precise identity of this factor and the molecular mechanism underlying hemolysis were only recently elucidated (99, 103). It was demonstrated that candidalysin was responsible for inducing red blood cell lysis, as *C. albicans ece1*∆/∆ and *ece1*Δ/Δ *+ ECE1*_Δ184–279_ mutant strains were unable to trigger this effect (103).

## CANDIDALYSIN IN IRON AND ZINC ACQUISITION

During tissue invasion and systemic infection, the host limits the availability of essential micronutrients, such as iron and zinc (100, 101, 104). To counteract these restrictions, *C. albicans* has developed a range of mechanisms to acquire vital micronutrients from host sources. For iron acquisition, the fungal adhesin and invasin Als3p binds to host ferritin, the primary epithelial iron storage protein, providing a critical iron source during epithelial cell invasion (105). Zinc availability is tightly regulated by the host during mucosal and systemic infection, with extracellular zinc depletion mediated by calprotectin, a host-derived zinc-binding protein (106). In response to zinc limitation, *C. albicans* activates a transcriptional zinc-starvation response, upregulating genes, such as *ZRT101* (encoding the cell-surface zinc importer Zrt101p) and *PRA1* (encoding the zinc-binding protein Pra1p), which are components of a so-called zincophore system (107).

Interestingly, zinc limitation and the resulting production of Pra1p may also be directly associated with the progression of VVC (108). Likewise, zinc deficiency is a significant risk factor for the development of systemic *C. albicans* infection in pediatric intensive care unit patients, with zinc supplementation being a recommended prophylaxis (109, 110).

*In vitro* studies have found that deletion of zinc acquisition genes, such as *ZRT101* and *ZRT2*, significantly reduces host-cell damage, which can be rescued by the exogenous addition of zinc. Notably, *C. albicans* exhibits a pronounced transcriptional zinc starvation response during *in vitro* growth, which is mitigated in the presence of intestinal epithelial cells, suggesting that the host cells serve as the primary zinc source during epithelial invasion (111). Deletion of the *ECE1* gene results in a marked increase in the transcriptional zinc starvation response in the presence of host cells, indicating that candidalysin-mediated host-cell membrane lysis is required to access intracellular zinc reservoirs. These findings highlight candidalysin as a vital factor enabling *C. albicans* to access host-derived micronutrients, thereby promoting fungal growth and driving pathogenicity. The interconnected regulation of fungal growth, candidalysin production, and zinc acquisition mechanisms may represent an example of predictive adaptation (112). In this process, the fungus upregulates zinc acquisition genes during hyphal formation, thus preparing for access to host-derived nutrients following cell invasion and candidalysin-induced damage.

## CANDIDALYSINS IN DIFFERENT SPECIES

Candidalysin was originally identified in the *C. albicans* strain SC5314 (13). Recently, a study identified orthologs of the *ECE1* gene and candidalysin in the pathogenic *Candida* species *C. dubliniensis* and *C. tropicalis*, thereby forming the first known family of cytolytic peptide toxins in fungi (113). Like the candidalysin from *C. albicans* SC5314, those from *C. dubliniensis* and *C. tropicalis* are flanked by lysine-arginine processing sites. Despite variations in amino acid sequence, all candidalysins share a conserved α-helical secondary structure and amphipathic properties. Interestingly, application of candidalysins from *C. dubliniensis* and *C. tropicalis* onto oral epithelial cells revealed significantly higher potency compared to *C. albicans*, inducing greater levels of cellular damage, calcium influx, and MAPK signaling (113). Additionally, candidalysins from *C. dubliniensis* and *C. tropicalis* were observed to permeabilize artificial planar lipid bilayers more rapidly than *C. albicans* candidalysin (113). Interestingly, despite this enhanced potency, *C. dubliniensis* and *C. tropicalis* exhibit reduced pathogenicity, which is likely attributed to their limited level of hypha formation and reduced *ECE1* expression. Notably, placing *C. dubliniensis* and *C. tropicalis ECE1* genes under the control of the *ECE1* promoter in *C. albicans* failed to induce epithelial cellular damage (113), highlighting the importance of additional regulatory factors in controlling *ECE1* expression, transcript stability, translation, folding, Ece1 processing, and secretion (89).

In addition to candidalysins identified in other *Candida* species, numerous candidalysin sequence variants have been identified within *C. albicans* (40, 114, 115) and in isolates of *C. dubliniensis* and *C. tropicalis* (115). Biophysical and biological characterization of these variants revealed differences in toxin potency and ability to activate host responses in comparison to their species-specific reference toxins (115). These findings highlight the critical role of specific amino acid residues in candidalysin activity.

## BEYOND CANDIDALYSIN: FUNCTIONAL INSIGHTS INTO NON-CANDIDALYSIN ECE1 PEPTIDES (NCEPS)

Most cell-damaging peptide toxins in biology are expressed as precursors. While candidalysin has similarities with other pore-forming peptide toxins, such as bee venom melittin (116), or phenol-soluble modulins of *Staphylococci* (117), the precursor structure of candidalysin is unusual. After translation, candidalysin is embedded within the polyprotein precursor Ece1p. This pre-pro-protein structure does not resemble the usual precursor of microbial peptide toxins (89). As discussed previously, it consists of a signal peptide, the precursor peptide of candidalysin (P3 in Fig. 1), and seven further non-candidalysin Ece1p peptides (NCEPs), each separated by lysine-arginine (KR) residues. Interestingly, a structurally similar yet unrelated polyprotein has been identified in the plant pathogenic fungus *Ustilago maydis*, containing several amphipathic peptides that function as hydrophobins (118). In Ece1p, the KR residues serve as processing sites for the Golgi-located proteases, Kex1p and Kex2p. Accurate kexin-mediated cleavage of Ece1p and the subsequent secretion of candidalysin into the extracellular space are critical for hyphal-driven epithelial damage (39). Interestingly, the pre-pro-protein structure is highly conserved among clinical isolates of *C. albicans* (89), suggesting that NCEPs play an important role in candidalysin delivery and *C. albicans* biology.

Numerous secreted proteins and peptides of eukaryotic cells are translated as precursors that require post-translational proteolysis by specific proteases. In many cases, removal of an N-terminal pro-domain is sufficient to activate the corresponding effector protein or peptide. This is also true for the production of many microbial pore-forming toxins (119–121). In these cases, a precursor is first produced and then processed to prevent self-toxicity. Based on the multiple examples of such precursors, it was initially hypothesized that the function of NCEPs might be to prevent self-toxicity in the fungus (89). However, *C. albicans* cells are not susceptible to candidalysin, and while the toxin integrates into artificial and human membranes, it does not integrate into *C. albicans* membranes. Moreover, single adjacent NCEPs that are fused to candidalysin are sufficient to prevent epithelial cell damage. Thus, the primary function of NCEPs and the Ece1p structure are not to prevent self-toxicity.

Conversely, it was shown that NCEPs are crucial for intracellular Ece1p folding and candidalysin secretion (89). The removal of multiple or single NCEPs or modifications to single peptide sequences caused an unfolded protein response, which in turn inhibited hyphal growth, epithelial invasion, and damage. This effect can be attributed to the extreme hydrophobicity of the candidalysin sequences, highlighting the necessity of shielding these sequences with NCEPs to prevent intracellular aggregation. Therefore, the Ece1p precursor is not required to block premature pore-forming toxicity but rather to prevent intracellular auto-aggregation of candidalysin sequences and guide the toxin through the secretory pathway, ensuring its controlled secretion into the extracellular space (89). It is likely that NCEPs are associated with the peptide toxin not only along the secretory pathway but also after its secretion to avoid extracellular aggregation and modulate its function (89). Indeed, the addition of synthetic NCEPs to synthetic candidalysin alters its properties and membrane integration dynamics. While NCEPs are not able to cause hemolysis alone, they still serve a function during hemolysis *in vivo*. Other NCEPs, particularly P7 and its derivative sub-fragment, are abundant in *C. albicans* supernatants (13, 39). Interestingly, derivative peptide fragments of P7 were able to increase the hemolytic activity of synthetic candidalysin, though the effect was modest (103). This suggests that while candidalysin is the only major hemolytic factor in *C. albicans*, P7 and other NCEPs might modulate its activities. Together, these data indicate that studies utilizing synthetic candidalysin may not entirely reflect the *in vivo* properties of the toxin.

## CANDIDALYSIN AS A THERAPEUTIC TARGET

Targeting candidalysin for therapeutic intervention offers a promising strategy to mitigate *C. albicans* infections without relying on traditional antifungals, which often leads to resistance. Neutralizing candidalysin activity has emerged as a potential therapeutic approach to prevent host cell damage and inflammation. For instance, the purinergic receptor antagonist pyridoxal-phosphate-6-azophenyl-2′,4′-disulfonic acid has been shown to reduce candidalysin-induced hemolysis by inhibiting its intercalation into synthetic membranes (103). Additionally, a llama-derived anti-candidalysin nanobody efficiently neutralized candidalysin-induced lysis of epithelial cells and significantly reduced tissue damage and inflammation *in vitro*. This nanobody-based approach could complement antifungal treatments like fluconazole to manage VVC symptoms and severity (66). Another promising avenue is the development of vaccines targeting candidalysin, designed to elicit protective immune responses akin to the NDV-3A vaccine, which targets Als3p (122–124). Furthermore, targeting candidalysin together with other recognized virulence factors, including adhesins and hydrolytic enzymes, may prove a useful combination therapy against *C. albicans* infections. However, it should be noted that candidalysin functions as both a virulence and avirulence factor, simultaneously causing cell damage while also inducing the recruitment of immune cells necessary for fungal clearance (16). This dual role underscores the challenge of targeting candidalysin therapeutically without compromising essential immune responses.

## CONCLUSION AND FUTURE PERSPECTIVES

Fungal infections are on the rise; they affect more than 1 billion people worldwide and kill 3.8 million people per year (1, 3). During the coronavirus disease 2019 (COVID-19) pandemic, we also witnessed the emergence of COVID-19-associated fungal infections, such as pulmonary aspergillosis and mucormycosis (125–127). COVID-19-associated candidiasis caused mainly by *C. albicans* and *C. auris* was the main COVID-19-associated fungal infection (126). A study spanning 2020–2021 in the United States revealed that critically ill hospitalized COVID-19 patients with fungal coinfections had a significantly higher mortality rate than patients with non-COVID-19 associated fungal infections (48.5% versus 12.3%, respectively) (127). These numbers highlight the importance of improving the surveillance, prevention, diagnosis, and treatment of fungal infections. In 2022, the World Health Organization published the first report on fungal priority pathogens. The report aims to guide research, development, and public health action and includes *C. albicans* in the “critical” pathogen group (highest), alongside *C. auris*, *Aspergillus fumigatus*, and *Cryptococcus neoformans*. The number of fungal infections is increasing, and no clinically approved vaccines are available against any fungal pathogen. Therefore, studying fungal infections is a moral imperative for this next decade.

Candidalysin plays a central role in the pathogenicity of *C. albicans*, acting as a multifaceted toxin that drives host cell damage and orchestrates complex immune responses. By damaging and activating host cells, such as epithelial cells, macrophages, and neutrophils, candidalysin contributes to fungal virulence and host defense, highlighting its dual role in host-pathogen interactions. Furthermore, its involvement in nutrient acquisition underscores its importance in supporting fungal growth and proliferation during infection. Only recently, it was observed that candidalysin is also a crucial commensal factor during gut colonization (128). Understanding the biology and activity of candidalysin is crucial to obtaining valuable insights into *C. albicans* pathogenesis and may open new avenues for therapeutic interventions targeting this critical virulence attribute.
